# Supplementary material for: Hydration product phase evolution and mortar strength development in alkali-activated slag and fly ash systems
Source: PLoS One. 2025 Dec 4;20(12):e0338119. doi: 10.1371/journal.pone.0338119 (PMC12677521; doi:10.1371/journal.pone.0338119)
Supplement: Attachment — Submitted filename: Review PLOS 1.pdf [file pone.0338119.s001.pdf]

Manuscript Click here to access/download;Manuscript;manuscript(1).doc

RESEARCHARTICLE

Study on the phase evolution of hydration products of alkali-activated solid waste materials and the strength of cemented sand

Zhuo Jin[1], Aimin Gong[1,*], Yier Huang[1],Yulin Peng[2] and Kang Yong[1]， Shanqing Shao[1,*]

1 College of Water Conservancy, Yunnan Agricultural University, Kunming 650201, China

2 Academic Affairs Office, Yunnan Agricultural University, Kunming 650201, China

* 13708457658@163.com; ssq87358764520@163.com

Abstract

Alkali-activated industrial solid waste is an ideal substitute material for Portland cement. Currently, the most commonly used industrial solid wastes are fly ash and slag. Therefore, the study investigated the mortar strength of slag and fly ash precursors under the action of different dosages (4%, 6%, and 8%) of two alkaline activators (NaOH and $Ca(OH)_2$) at standard curing ages of 3, 7, and 28 days. The hydration product phase evolution of the two industrial solid wastes were analyzed through scanning electron microscopy (SEM), X-ray diffraction (XRD), Fourier-transform infrared spectroscopy (FTIR), isothermal calorimetry, and backscattered electron imaging (BSE). The micromorphology, phase composition, hydration process, and porosity of the hydration products were characterized at the micro level. The results show that the slag system is more sensitive to NaOH, and at a dosage of 8% NaOH, its hydration reaction is sufficient, with abundant hydration products and a denser pore structure, leading to a significant improvement in mechanical properties. The fly ash system is more sensitive to $Ca(OH)_2$, and at a dosage of 6% $Ca(OH)_2$, the hydration reaction of fly ash is sufficient. Appropriately increasing the Ca/Si ratio results in higher polymerization of the hydration products, significant optimization of the pore structure, and optimal me-chanical properties. However, excessive $Ca(OH)_2$ inhibits the hydration reaction of fly ash, leading to deteriorated pore structure and reduced strength. Comprehensive analysis indicates that the performance of alkali-activated geopolymer cementitious materials can be directionally regulated through the appropriate design of activator type and dosage, providing theoretical and technical support for improving the utilization of alkali-activated geopolymer cementitious materials.

Key words: slag; Fly ash; Setting time; Strength; Hydration; microstructure

Introduction

With the development of the water conservancy construction industry, the cement has become one of the largest amount of building materials today, however, the cement production process has been accompanied by high emissions, high energy consumption and other issues (carbon emissions account for about 7%-8% of the total global emissions, the cement production process needs to consume a large amount of limestone and fuels, and at the same time, it will also produce a large amount of dust and wastewater) [1,2], which not only creates a serious load on the environment. This is not only a serious load on the environment, but also not conducive to the realization of China's dual-carbon goals and the green, healthy and sustainable development

of the water conservancy construction industry, in this context, alkali excitation of solid-waste cementitious materials came into being, which is a class of green geopolymer cementitious materials that can be used to replace cement by using alkaline exciters to stimulate the volcanic ash activity of industrial solid waste (slag, fly ash) or natural silicate minerals (biotite kaolinite, bentonite) [3,4]. The material is a green geopolymer cementitious material that can replace cement [3,4]. Compared with ordinary silicate cement, alkali-inspired solid waste cementitious materials have many significant advantages, such as wide material sources, no need for high-temperature calcination, low carbon emissions, low energy consumption, low exothermic hydration, which can effectively reduce the generation of temperature cracks, good cementing properties, excellent durability can maintain long-term stability in complex environments, etc., so in the construction field, road and transportation engineering, bridge and tunnel engineering, water conservancy engineering, Marine engineering, repair and reinforcement engineering, 3D printing technology, and environmental protection applications (curing heavy metals) and other fields have a very wide range of application prospects [5-7].

Slag produced in the process of blast furnace ironmaking [8], coal-fired power generation process of fly ash [9] is China's current emissions of industrial by-products, of which the main components of slag are silicate and alumina, the main component of fly ash is silica, alumina and calcium oxide, etc., which endows slag and fly ash with a good volcanic ash activity, so that it can be used as a high-quality auxiliary cementitious materials, but the current comprehensive The utilization rate is low [10], so in order to realize the bulk utilization of these solid wastes, reduce the accumulation of waste and pollution of the environment, and to provide a scientific basis for its application in engineering, it is particularly important to study its performance under different conditions, in which the type of alkaline exciter and the amount of alkaline exciters affect the alkaline-excited cementitious materials are important factors, therefore, many researchers have done a lot of research on the performance of alkaline exciters. Therefore, many researchers have done a lot of research on their properties under the action of alkaline exciters. Sun Kaiqiang [11] and Li Ping [12] showed that slag and fly ash showed high activity under the action of alkaline exciters, which provided powerful conditions for the development of mechanical properties, but the type and concentration of alkaline exciters were important factors affecting the mechanical properties of geopolymers; Liu Yulin [13] showed that NaOH could effectively stimulate the activity of fly ash, so that the compressive strength of cement-fly ash matrix could reach 4.21Mpa, and the compressive strength of cement-fly ash matrix could reach 4.21Mpa, and the compressive strength of cement-fly ash matrix could reach 4.21Mpa. can reach 4.21Mpa, which indicates that NaOH has a unique advantage in stimulating the activity of fly ash, and can significantly improve the mechanical properties of cementitious materials; Qiao Longteng [14] showed that compared with Na2SiO3, the microstructure of cement-fly ash based cementitious body is more dense under the action of NaOH, and the strength of the filling body is obviously improved, and the improvement effect on 28d compressive strength is the most prominent, which indicates that the improvement effect of NaOH is the most important factor for the mechanical properties of cementitious materials. is most prominent, which indicates that NaOH has a unique advantage in stimulating the activity of fly ash, and can significantly improve the mechanical properties of cement-based materials; the study of Han Xiangming [15] show that slag replaces part of the cement to improve the microstructure of cement mortar, reduces microcracks and pores, improves the splitting tensile strength of the matrix, and improves the

ductility and deformability of cement-concrete, which suggests that slag in cement-based materials has good compatibility and can improve the mechanical properties of the material by improving the microstructure; Yu Qi [16] investigated the effects of NaOH and water glass on the compressive strength and resistance to chloride ion erosion of slag concrete, and the results showed that the compressive strength of water glass-excited specimens was higher than that of NaOH-excited specimens, but the resistance of NaOH-excited specimens to chloride ion erosion was higher than that of water glass-excited specimens; The study of Zuo [17] showed that the degree of reaction of slag gradually increased when the amount of NaOH was from 4%-8%.

In summary, different exciters have different effects on the performance of alkali-excited cementitious materials, and it is necessary to select the appropriate exciters according to the specific needs of the application, the current research on alkali-excited solid-waste cementitious materials has achieved certain results, but there are still some problems that need to be further explored, and the current research mainly focuses on clarifying the strength of the specimen when NaOH, Na_2SiO_3 is used in single doping or compound doping, and the strength of the test piece and relatively little research on the evolution of its hydration product phase. The evolution of hydration product phase is an important factor affecting the performance of alkali-inspired solid waste cementitious materials, and it is of great significance to understand the evolution of hydration product phase for optimizing the performance of the materials. Therefore, this paper compares the hydration product phase evolution of NaOH, $Ca(OH)_2$ and its different dosage on slag and fly ash, and explores its mechanism in depth to provide a solid theoretical basis for the utilization of industrial solid waste resources.

Materials and Methods

materials

The slag was S95 granulated blast furnace slag produced by Gongyi Longze Water Purification Materials Co., Ltd, with specific surface area 429.00 m^2/kg, flow ratio ≥95%, 7d activity index ≥70%, 28d activity index ≥95%, and density ≥2.8 g/cm2;

Fly ash adopts the first-grade fly ash produced by Gongyi Run Refractory Material Co;

Alkaline exciter: Hui-lime brand lime powder produced by Yixian Huangbaozhu Daily Chemical Products Co., Ltd, with CaO content of 93.45%， solid sodium hydroxide with purity of 98% produced by Tianjin Zhiyuan Chemical Reagent Co;

Fine aggregate: China ISO standard sand produced by Aceo Standard Sand Co;

Water: laboratory tap water

Table 1 Chemical composition of geopolymers

constituent	Compositions/%								
	CaO	SiO2	Al_2O_3	SO_3	Fe_2O_3	MgO	Na_2O	K_2O	other
Slag	34	34.5	17.7	1.64	1.03	6.01	—	—	5.12
Fly ash	4.5	45.1	36.8	1.2	0.85	—	—	—	11.55

Mixes design

In order to exclude the influence of exothermic alkaline exciters on the test results, the NaOH solution and $Ca(OH)_2$ solution were prepared 24h in advance and sealed for storage. The mixing proportions of the different cementitious systems are shown in Table 2.

Table 2 Mixture proportions of the different cementitious systems

sample	Materials	Types of alkaline activators	Exciter content of alkaline /%
4SN	Slag	NaOH	4
6SN	Slag	NaOH	6
8SN	Slag	NaOH	8
4SC	Slag	$Ca(OH)_2$	4
6SC	Slag	$Ca(OH)_2$	6
8SC	Slag	$Ca(OH)_2$	8
4FN	Fly ash	NaOH	4
6FN	Fly ash	NaOH	6
8FN	Fly ash	NaOH	8
4FC	Fly ash	$Ca(OH)_2$	4
6FC	Fly ash	$Ca(OH)_2$	6
8FC	Fly ash	$Ca(OH)_2$	8

Note: The amount of alkaline stimulant is determined according to the total mass ratio of cementitious materials.4, 6 and 8 denote the mass fraction of alkaline stimu-lant dosage, S denotes slag, F denotes fly ash, N denotes NaOH, C denotes $Ca(OH)_2$, NN denotes NaOH+Na2SiO3, 6SN denotes the addition of 6% NaOH to the slag, and so on.

methods

According to 《Test Method for Standard Consistency, Setting Time and Stability of Cement》 (GB/T 1346-2011), the initial setting time and final setting time of each group of specimens were determined by using Vickers meter;

According to 《Test Method for Strength of Cementitious Sand (ISO Method)》 (GB/T 17671-2021), prepare cementitious sand specimens of slag system and fly ash system, according to the proportion (shown in Table 2), weigh the corresponding qual-ity of the material and put it in the cementitious sand mixer and mix it evenly, then prepare the cementitious sand specimens of 40mm*40mm*160mm, and mold 9 speci-mens for each group of proportion, After 24h of natural maintenance at room temper-ature, the specimens were demolded and put into the standard maintenance box (temperature ah (20±2)℃, relative humidity not less than 95%) to be raised to 3d, 7d, 28d, respectively, to determine the flexural strength and compressive strength of the corresponding age of the cement sand specimens.

The crushed specimens in the compressive strength test were placed in anhydrous ethanol to terminate hydration, and after 3d, they were taken out and dried, and a part of them was used for SEM scanning electron microscope analysis, and the other part was ground and passed through a sieve of 0.075mm, and was used for XRD and FTIR analyses, in order to characterize the microstructures of the corresponding test groups, and the types of hydration products.

The heat of hydration was analyzed by using TAM Air isothermal calorimeter produced by TA, USA to determine the rate of hydration exotherm and the amount of exotherm of slurry 3d.

Backscattered image analysis (BSE-IA method) was performed on the BSE pictures of the mastic sand specimens of each test group with a curing age of 28 d with the help of Image-Pro Plus 6.0 image recognition software, so as to characterize the pore struc-ture of the slag-based mastic sand specimens and fly ash-based mastic sand specimens.

Results and discussion

Setting time

Figs 1 and 2 demonstrate the effect of NaOH and $Ca(OH)_2$ on the setting time of slag system and fly ash system, respectively. By comparing Figs 1 and Figs 2, it can be found that the setting time of the slag system is significantly lower than that of the fly ash system, which is mainly due to the lower degree of polymerization of the vitreous structure in the slag [18], which is easier to dissolve and generate hydration products under the action of alkaline exciters; moreover, the setting time of the slag system is significantly shorter under the action of NaOH as compared to that of the experimental group with the addition of $Ca(OH)_2$, and the The setting time of the fly ash system was significantly prolonged, which indicated that the slag showed strong sensitivity to NaOH, while the fly ash showed strong sensitivity to $Ca(OH)_2$. Analyzing the reasons, this may be due to the fact that $Ca(OH)_2$ added to make the high-calcium type slag matrix hydration system in the Ca/Si further increase and thus inhibit the dissolution of the slag particles, and for low-calcium type fly ash, the hydration system in the Ca/Si ratio increased, thus suppressing the dissolution of slag particles. The in-crease of Ca/Si ratio is conducive to promote the hydration reaction to proceed and generate hydration products [19,20], thus shortening the setting time.

It can also be seen from Figs.1 that when the dosage of NaOH and $Ca(OH)_2$ is increased from 4% to 8% respectively, the setting time of slag system is shortened, in-dicating that the alkaline enhancement of the two alkaline exciters is conducive to in-crease the dissolution rate of the active ingredient in the slag, and to accelerate the hardening of the gelatinization system. From Fig. 2, NaOH dosage increased from 4% to 8%, the coagulation time of fly ash system was shortened, and $Ca(OH)_2$ dosage increased from 4% to 8%, the setting time was shortened and then prolonged, indicat-ing that NaOH alkaline enhancement is conducive to improving the dissolution rate of the active ingredient in the fly ash, The appropriate increase in the dosage of $Ca(OH)_2$ is beneficial to provide suitable alkaline conditions to promote the dissolution of fly ash particles, while too much dosage will inhibit the dissolution of the active ingredi-ents, thus prolonging the setting time. In summary, by adjusting the type and dosage of the exciter, the coagulation performance of these materials can be optimized to meet different engineering needs.

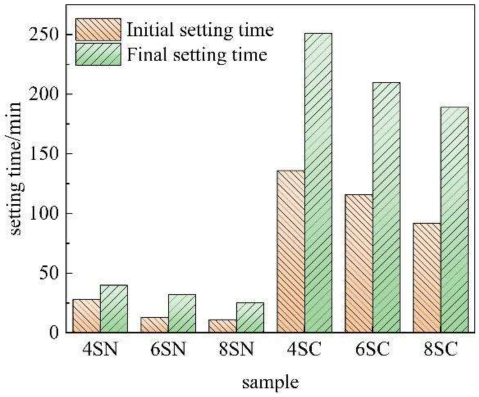

Fig 1. Slag setting time

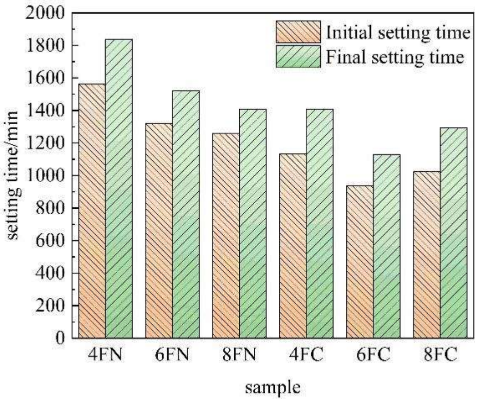

Fig 2. Fly ash setting time

Compressive strength and flexural strength

Fig 3 and Fig 4 shows the change rule diagram of compressive strength and flexural strength of slag system under the action of NaOH and $Ca(OH)_2$. As can be seen from the Fig 3, slag shows

higher compressive strength and flexural strength under the ac-tion of NaOH, which is significantly better than that of the test group adding $Ca(OH)_2$, indicating that the hydration rate of slag is faster under the action of NaOH, and the generation of hydration products is increased, which shows higher sensitivity to NaOH, and with the increase of NaOH doping from 4% to 8%, the compressive strength and flexural strength of the slag system are increased, which was attributed to the increase of alkalinity, resulting in the decrease of bonding energy between Si-O and Al-O bonds in the slag [21], which led to the dissolution of more $[SiO_4]^{4-}$ and $[AlO_4]^{5-}$ in the vitreous structure, which reacted with Na^+ and Ca^{2+} in the solution to form a gel[22,23], and compacted the matrix, and the formation of more compressive products, which densifies the matrix pore structure and thus improves the strength; when $Ca(OH)_2$ doping increases from 4% to 8%, the compressive strength gradually increas-es while the flexural strength gradually increases at 3d and 7d, and shows a tendency to increase first and then decrease at 28d.

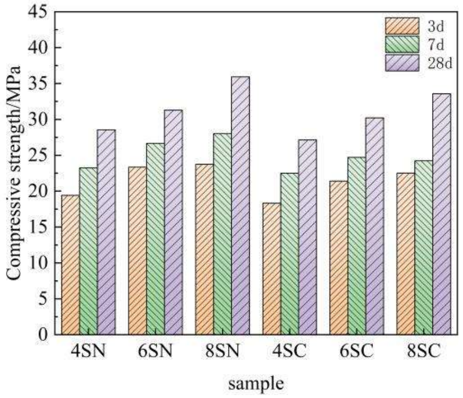

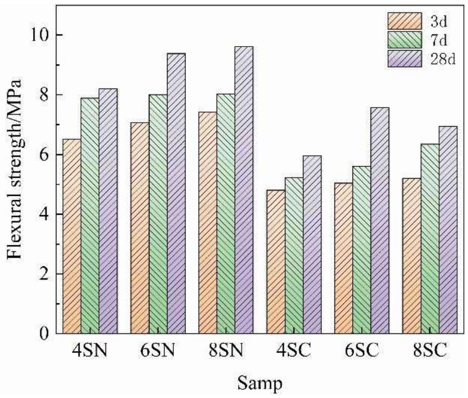

Fig 3. Compressive strength of slag **Fig.4 The flexural strength of slag**

Fig 5 and Fig 6 shows the change rule diagram of compressive strength and flexural strength of fly ash system under the action with NaOH and $Ca(OH)_2$. From the Fig, it can be seen that the strength of fly ash under the action of $Ca(OH)_2$ in the cemented sand are significantly higher than that of the test group with the addition of NaOH, which indicates that $Ca(OH)_2$ can better stimulate the activity of fly ash, and the anal-ysis of the reason may be due to the addition of $Ca(OH)_2$ improves the Ca/Si ratio of the hydration system, promotes the rate of the hydration reaction, and thus promotes the development of the strength and the $Ca(OH)_2$ provides the Ca^{2+} can replace the Na^+ in the hydrated sodium silica-aluminate (N-A-S-H) gel, generating a higher density of hydrated calcium silica-aluminate (C-A-S-H) gel, and the two kinds of gels intertwined with each other, resulting in the further enhancement of the strength and the rate of growth of the strength increased significantly. When the NaOH dosage was increased from 4% to 8%, the compressive and flexural strengths were gradually enhanced, in-dicating that the increased alkalinity of NaOH was beneficial to the promotion of the hydration of fly ash particles; when the $Ca(OH)_2$ dosage was increased from 4% to 8%, the compressive and flexural strengths were firstly increased and then decreased, which was attributed to the excessive alkalinity of $Ca(OH)_2$ dosage of 8% as well as the Ca/Si ratio exceeded the appropriate range. Thus the fly ash vitreous structure depol-ymerization was inhibited.

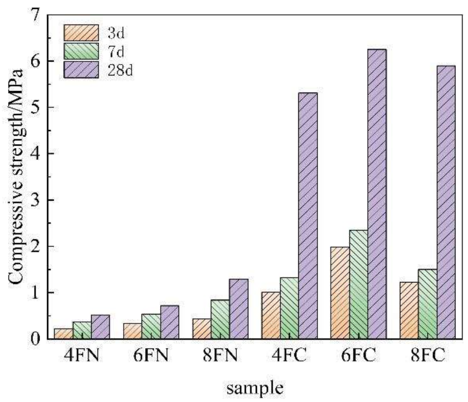

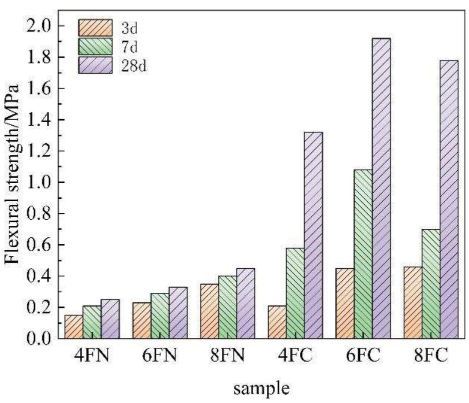

Fig.5 The compressive strength of fly ash **Fig.6 The flexural strength of fly ash**

Sensitivity analysis

In the slag system with 4SN group as the reference group, in the fly ash system with 4FN group as the reference group, to clarify the sensitivity change rule of the slag system and fly ash system to the type and dosage of exciter,as shown in table 3.

Table 3 Sensitivity coefficients

Sample	Compressive strength sensi-tivity factor			Flexural strength sensitivity factor		
	3d-7d	7d-28d	average	3d-7d	7d-28d	average
4SN-6SN	0.11	0.23	0.17	0.12	0.61	0.37
4SN-8SN	0.43	1.11	0.77	0.32	0.99	0.65
4SN-4SC	0.07	0.24	0.15	-0.50	-0.14	-0.32
4SN-6SC	0.11	0.47	0.29	-0.33	0.96	0.32
4SN-8SC	0.25	0.96	0.60	-0.21	0.26	0.02
4FN-6FN	0.40	0.20	0.30	0.17	0.25	0.21
4FN-8FN	1.73	2.00	1.87	0.33	0.50	0.42

From the table, it can be seen that the sensitivity of slag system to NaOH is high, especially the flexural strength is significantly improved, but the sensitivity to $Ca(OH)_2$ is low, and the sensitivity coefficient is negative compared with 4SN; while the sensi-tivity of fly ash system to $Ca(OH)_2$ is very high, and the compressive strength and flexural strength are significantly improved, much higher than that of $Ca(OH)_2$ system. The slag system is insensitive to changes in the dosage of exciter, while the fly ash sys-tem is very sensitive to changes in NaOH dosage. Therefore, the slag system is more suitable for the use of NaOH as an exciter, while the fly ash system is more suitable for the use of $Ca(OH)_2$, and the mechanical properties can be optimized by increasing the dosage appropriately.

SEM analysis

Fig 7 shows the SEM pictures of the slag system at each age of maintenance. As can be seen from the Fig, a large number of long-chained and loose and porous C-(A)-S-H gels covering the surface of unhydrated slag particles can be observed in the slag matrix with 6% $Ca(OH)_2$ added at 3d (shown in Fig a), and these hydration products dehydrated and condensed to form a more continuous microstructure when maintained until 28d (shown in Fig c), but larger pores can still be clearly observed; When the dosage of $Ca(OH)_2$ was 8%, the hydration products of reticulated structure were not observed when curing to 3d (shown in d), but a large number of flocculent C-(A)-S-H gel and needle and rod calcium alumina (AFt) were generated, and the number of

calcium alumina was increased when curing to 7d (shown in e), which may be attributed to the fact that the addition of calcium hydroxide to the slag made the concentration of Ca^{2+} in the hydration system rise, and the concurrently Alkalinity is enhanced, the Ca^{2+} dissolution rate in the geopolymer is accelerated, thus promoting the generation of AFt. The expansion of AFt can inhibit the contraction and defor-mation of the collodion, which explains why the strength of the 8SC group is higher than that of the 6SC group, and the hydration products increase significantly when maintained up to 28d (shown in f), filling the pore structure and forming a more ho-mogeneous, dense, and continuous colloid[12], thus improving the mechanical proper-ties. Under the action of 6% NaOH, the unhydrated slag particles were obviously re-duced at 3d (shown in g), and the generated flocculent C-(A)-S-H and lamellar rhom-bohedral calcium zeolite lapped together, with a loose structure, and at 7d (shown in h), the generated C-(A)-S-H gel and rhombohedral calcium zeolite interspersed with each other, which can play a role in enhancing the strength, but a large number of large pores are still seen, and the conservation of By 28d (shown in i), the hydration products gradually increased and became larger, the rod-like structure disappeared, and a dense microstructure was formed; compared with 6% NaOH doping, the polymerization degree of hydration products was higher under the action of 8% NaOH by the time of maintenance up to 3d (shown in j), and a more dense microstructure was formed by the time of 7d (shown in k), and by the time of maintenance up to 28d (shown in L), these hydration products further dehydrated and condensed, and the microstructure was more dense.The microstructure of 8SN was the densest at 28d, in-dicating that the excitation effect of NaOH was better than that of $Ca(OH)_2$ and the 8% NaOH dosing had the best effect on the slag.

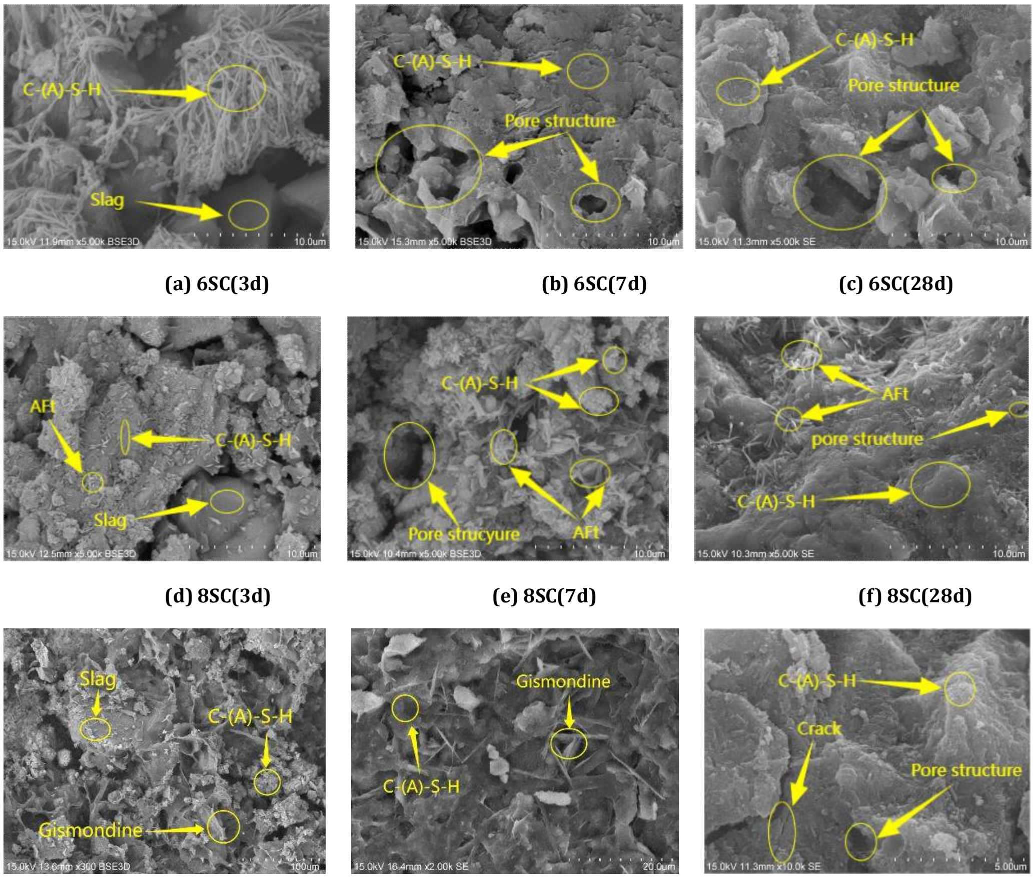

(a) 6SC(3d) **(b) 6SC(7d)** **(c) 6SC(28d)**

(d) 8SC(3d) **(e) 8SC(7d)** **(f) 8SC(28d)**

(g) 6SN(3d) (h) 6SN(7d) (i) 6SN（28d）

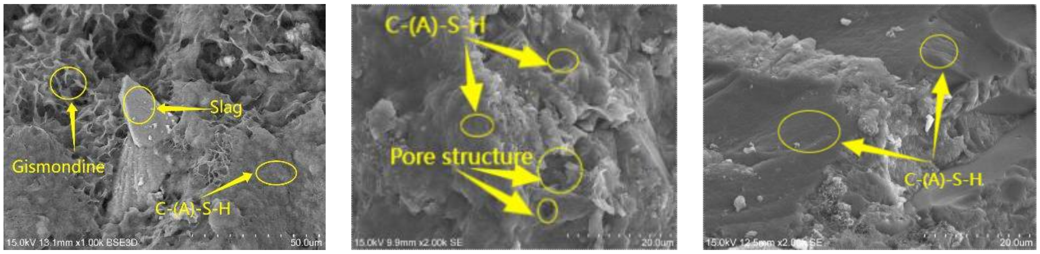

(j) 8SN(3d) (k) 8SN(7d) (L) 8SN(28d)

Fig 7. SEM images of slag under the action of 6%, 8%NaOH and Ca(OH)2 respectively SEM

Fig 8 shows the SEM pictures of the fly ash system at each age of maintenance. As can be seen in the Fig, when $Ca(OH)_2$ was used as an exciter, a large number of fly ash particles not involved in hydration and agglomerated together were still clearly observed on the micrographs when the fly ash specimens were maintained up to 3d and 7d, and only a small amount of loose and porous flocculent (a, b) or reticulated (d, e) C-(A)-S-H and N-A-S-H attached to the surface of the fly ash particles or filled in between fly ash particles, and when maintained up to 28d (c, f), hydration products increased, which attached the unhydrated particles to the fly ash particles. or filled between the fly ash particles, and when maintained until 28d (c and f diagrams), the hydration products increased, completely wrapping the unhydrated fly ash particles and cementing them with the hydration products away from the surface of the fly ash particles, resulting in an increase in the continuity and polymerization of the test blocks [24], but the contours of the incompletely hydrated fly ash particles could still be observed, with the microstructure of the 6FC group at 28d (c diagram) being the most dense . Fly ash maintained under the action of sodium hydroxide until 28d (g and f di-agrams) can be seen in the microstructure there are still a large number of unhydrated fly ash particles exist, indicating that NaOH as an excitation agent excitation of fly ash, the excitation effect of fly ash is not obvious, only a small amount of flocculated hy-dration products generated, can not play a better role in cementation, which leads to poor mechanical properties. From the above analysis, it can be seen that the micro-structure of fly ash microsand is optimal when the dosage of $Ca(OH)_2$ is 6%.

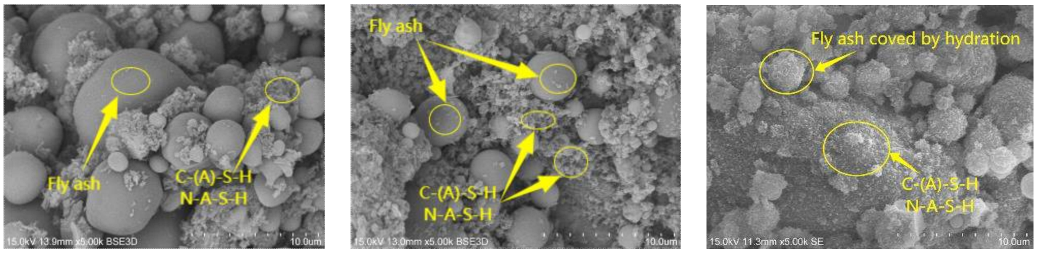

(a) 6FC(3d) (b) 6FC(7d) (c) 6FC(28d)

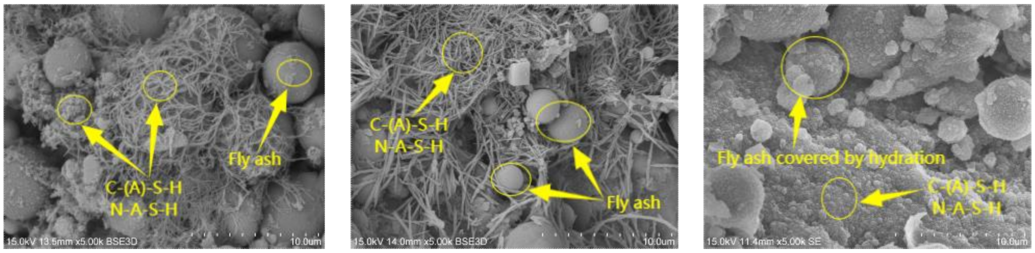

(d) 8FC(3d) (e) 8FC(7d) (f) 8FC(28d)

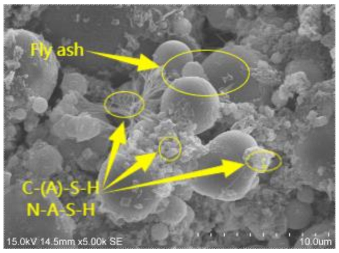

(g) 6FN（28）

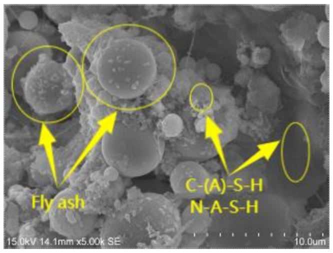

(h) 8FN（28）

Fig 8. SEM diagram of fly ash under the action of 6%, 8%NaOH and $Ca(OH)_2$ respectively

XRD analysis

In order to understand the effect of different types and dosages of alkaline exciters on the physical phase evolution of slag and fly ash hydration products, the specimen samples with a curing age of 28 d were characterized using XRD, as shown in Fig 9 and Fig 10 Since C-(A)-S-H gel and N-A-S-H gel are amorphous substances, their diffraction peaks could not be seen in the Fig. The diffraction peaks in alkali-excited slag system mainly include the diffraction peaks of quartz and rhombohedral calcium zeolite in the raw material and the diffraction peaks in alkali-excited fly ash system mainly in-clude the diffraction peaks of mullite, quartz and Ca_2CO_3 generated by carbonization in the raw material. However, there are some differences in the intensity of the corre-sponding diffraction peaks, indicating that different types and amounts of alkali excit-ers will have different effects on the hydration reaction of raw materials.

As shown in Fig 9, in the slag system, the diffraction peaks of quartz in 8SN were lower and those of rhombohedral calcium zeolite were higher compared with 6SN, indicating that the increase of NaOH doping, the generation of C-(A)-S-H gel and other hydration products gradually increased; 8SC and 6SC comparison, 8SC in the quartz diffraction peak value is lower, the diffraction peak value of rhombohedral cal-cium zeolite is higher, indicating that the increase in the dosage of $Ca(OH)_2$ is also conducive to promote the depolymerization of slag particles condensation. Among them, the 8SN group has the lowest intensity of quartz diffraction peaks and the strongest intensity of rhombohedral calcium zeolite diffraction peaks, indicating that 8% NaOH doping is more favorable to the dissolution of the glassy phase structure of the slag, and promotes the generation of C-(A)-S-H gels and rhombohedral calcium zeo-lites. The diffraction peak of $Ca(OH)_2$ was also observed in 8SC and 6SC, indicating that $Ca(OH)_2$ was not completely involved in the hydration or generated by the reac-tion between CaO and water in slag, and the diffraction peak of AFt was observed at 11.44[illegible], which may be generated by the reaction between free Ca^{2+} and $[AlO_4]^{5-}$ in the hydration system and sulfate in the gelling system. sulfate in the gelling system.

As can be seen from Fig 10, the diffraction peak intensities of quartz and mullite in 6FC and 8FC are significantly lower than those of 6FN and 8FN, indicating that it is more favorable for fly ash particles to dissolve under the action of $Ca(OH)_2$ to generate C-(A)-S-H gel and N-A-S-H gel.The diffraction peak intensities of quartz and mullite in 8FN are lower compared to that in 8FN compared to that in 6FN, indicating that the NaOH increase in the dosage can promote the participation of fly ash particles in hy-dration and generate a large number of N-A-S-H gels; compared with 8FC and 6FC, the diffraction peak intensities of quartz and mullite in 8FC are relatively high, indi-cating that an increase in the dosage of $Ca(OH)_2$ up to 8% inhibits the further

hydra-tion of fly ash particles and affects the generation of amorphous C-(A)-S-H gel and N-A-S-H gel, which mainly provide strength, thus resulting in a deterioration trend in strength. This results in a deterioration trend in strength. Among them, the diffraction peak peaks of quartz and mullite were the weakest in 6FC, suggesting that 6% $Ca(OH)_2$ can better stimulate the activity of fly ash. Diffraction peaks of Ca_2CO_3 were also seen in 8FC and 6FC, indicating that carbonization occurred in the specimen, but the dif-fraction peak of Ca_2CO_3 in 8FC was stronger than that in 6FC, which may be due to the fact that the large dosage of $Ca(OH)_2$ did not participate completely in the hydration process of fly ash, and the remaining portion of it carbonized after contacting with air.

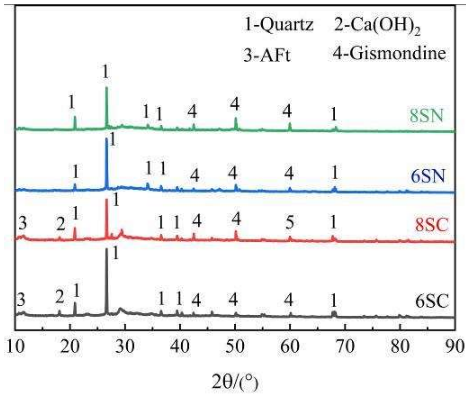

Fig 9. XRD patterns of slag

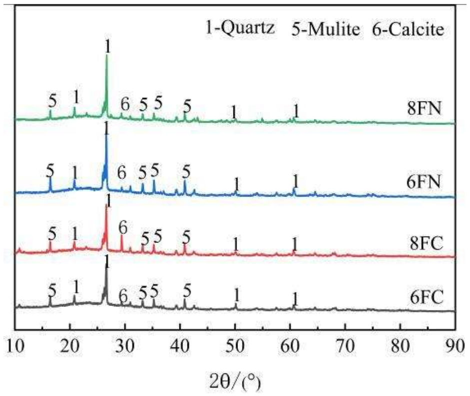

Fig 10. XRD patterns of fly ash

FIRT analysis

Fig 11 and Fig 12 shows the FTIR profiles of each specimen at 28 d of conditioning, and the samples were analyzed by FTIR to clarify the type of chemical bonding in the samples, and thus determine the type of hydration products. From the Fig, it can be seen that the vibrational peaks at 1300-850 cm^{-1} belong to the absorption peaks of Si-O-T (T is Si or Al) [25]; the telescopic vibrational peaks near 743 cm^{-1} belong to the Si-O-Si; and the characteristic peaks near 1382 cm^{-1} belong to the O-C-O, which indicates that the specimens were carbonized [25]. From the Fig, it can be seen that the slag system and the fly ash system have similar absorption bands under the condition of different types and dosages of alkaline exciters, which indicates that there is no significant change in the type of hydration products of the same material under the action of different types and dosages of alkaline exciters [26].

As can be seen from Fig 11, the intensity of the absorption peak peaks of the 8SN and 6SN groups is significantly stronger than that of the 8SC and 6SC groups, indicat-ing that NaOH can improve the surface activity of slag more efficiently; comparing the 8SN and 6SN groups, the peak values of the transmittance peaks of the Si-O-T bonds and Si-O-Si bonds of the 8SN group are larger but shifted to a lower wavelength, which suggests that the hydration reaction is more advanced with the addition of 8% NaOH. The C-(A)-S-H gel generated increases but the degree of polymerization de-creases, however, at this time, a large number of C-(A)-S-H gel contribution to the strength can effectively offset the negative impact of the decrease in the degree of polymerization, which makes the 8SN show relatively high mechanical properties; comparing the 8SC and 6SC groups, the peak value of the transmission peaks of the Si-O-T bond in the 8SC is also shifted from a low wave number to a higher wave num-ber, which indicates that a higher degree of polymerization and a more structural structure are generated. a gel with higher degree of polymerization and more stable structure [27].

From Fig 12, it can be seen that the transmission peak values of Si-O-T and Si-O-Si bonds in 8FN are larger than that of 6FN, and the transmission peak values of Si-O-T and Si-O-Si bonds in 6FC are larger than that of 8FC, indicating that the in-crease of NaOH doping promotes the depolymerization of the glassy structure of the fly ash, and facilitates the generation of the N-A-S-H gel, while the increase of $Ca(OH)_2$ doping will inhibit the depolymerization of fly ash and hinder the generation of N-A-S-H gel and C-(A)-S-H gel; compared with the 6SN and 8SN groups, the peaks and wave numbers of Si-O-T bond and Si-O-Si bond in 6SC and 8SC were larger, indi-cating that more hydration products were generated and the degree of polymerization was higher, which also further illustrated the strong sensitivity of fly ash to $Ca(OH)_2$, which was in line with the strength and condensation time results correspond.

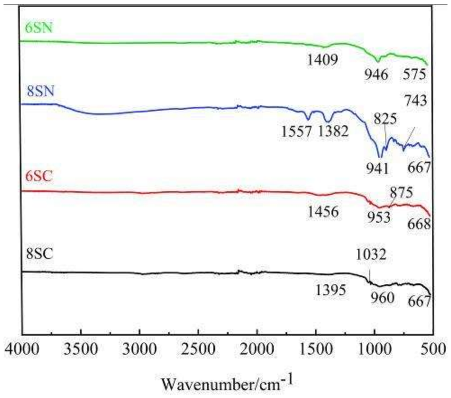

Fig 11. FTIR diagrams of slag

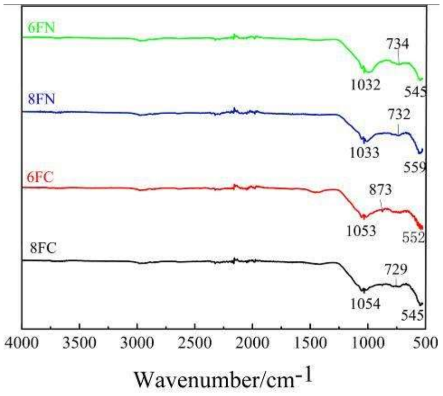

Fig 12. FTIR diagrams of fly ash

Hydration characterization

The hydration exothermic rate and the accumulation curve of hydration exother-mic quantity can react to some extent to the degree of hydration reaction of solid waste [28] and the phase evolution law, and the whole hydration process can be divided into five phases, i.e., the pre-induction period, the induction period, the acceleration period, the deceleration period and the stabilization period [29]. Fig 13 and Fig 14 shows the calorimetric curves of slag under the action of 8% NaOH and fly ash under the action of 6% $Ca(OH)_2$ for 72h.

From Fig 13, it can be seen that there are two hydration exothermic peaks in the hydration exothermic rate curves. The first exothermic peak corresponds to the disso-lution of geopolymer particles, i.e., the hydration reaction that occurs immediately af-ter the contact of geopolymer particles with water in the pre-induction period, in which the exchange of substances between the solid phase and the liquid phase occurs in the form of ions, and a layer of solid-liquid mixed phase is formed on the surface of the geopolymer particles; and the second exothermic peak corresponds to the rate of the generation of the hydration products, such as C-(A)-S-H, N-A-S-H and so on [30].

The first hydration exothermic peak of slag appeared at about 0.2h, when the slag particles dissolved rapidly and the exothermic rate was the fastest, and the second characteristic peak appeared at about 13h, when the ion concentration in the hydra-tion system reached the critical value, and the ions reacted rapidly with each other to generate the hydration products, such as C-(A)-S-H gel, etc.; the first hydration exo-thermic peak of fly ash appeared at about 0.6h, and the second peak of exothermic hy-dration appeared at about 16.6 h, but the rate of exothermic hydration was almost close to 0 at about 40 h. It can be seen in Fig 14 that the cumulative exothermic amount increased with the prolongation of time, which indicated that the hydration

products gradually increased, and their volume gradually increased; however, the in-crease of the cumulative exothermic amount gradually slowed down, especially after the entry of the stabilization period, and the increase was further reduced, and the rate of dissolution of ground aggregate particles as well as the generation of hydration products was slowed down, because at this time, the hydration reaction mainly relies on the movement and rearrangement of ions in the solid phase to realize the reaction rate is very slow.

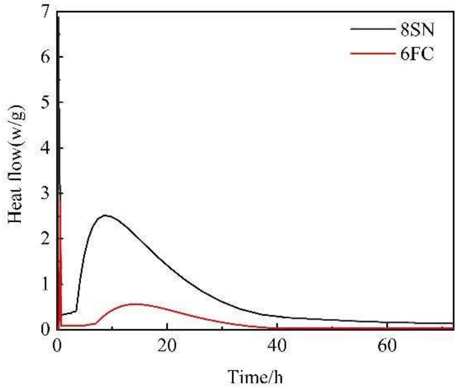

Fig 13. hydration heat release rate

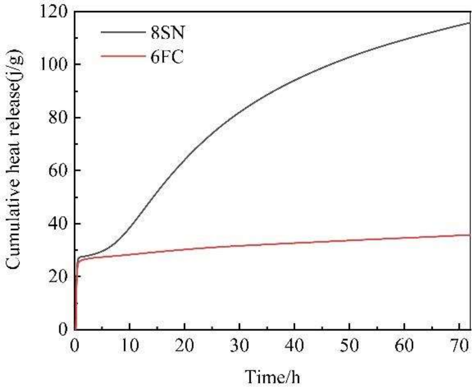

Fig 14. Cu-mulative heat release

Pore physical phase distribution and pore structure characteristics

Fig 15 shows the physical phase distribution of the pores of the grits of the slag system and fly ash system, with the pores marked in red, and Table 3 shows the char-acteristic parameters of the pore structure of the grits of the slag system and fly ash system. Comparing the backscattering diagrams of 6SN (a) and 8SN (b), there are more pores in 6SC and the phenomenon of large concentrated dispersion occurs, and at the same time, it can be seen from Table 3 that, compared with the 6SN group, the porosity in 8SN decreases, the number of pores decreases, the maximum diameter of pores de-creases, the total area of pores increases, and the number of small pores accounts for an increase in the ratio, which means that the effect of 8% NaOH on the slag is better than the 6% NaOH. Compared with 6SC (c), the pores in 8SC (d) are relatively less and evenly distributed, as shown in Table 3, the porosity of 8SC is 14.56% lower than that of 6SC, the number of pores is significantly reduced, and the maximum pore diameter is significantly reduced, which indicates that the effect of 8% $Ca(OH)_2$ on the slag is better than that of 6% $Ca(OH)_2$. 8SN, 6SN, 6SC, and 8SC, the 8SN has the lowest po-rosity, the fewest pores, the smallest maximum diameter of pores, and the most uni-form distribution, which indicates that the reaction of slag under the action of 8% NaOH is more complete, and the generation of hydration products is more, which can fill the pore structure better. Comparing the graphs of 6FC (e) and 8FC (f), it can be observed that 6FC has fewer pores and weaker concentration and dispersion in multi-ple regions. Additionally, Table 3 shows that the porosity of the 6FC group is relatively low, with fewer pores, smaller maximum pore diameter, larger total pore area, and a higher proportion of small pores. This indicates that the addition of 6% $Ca(OH)_2$ can promote more complete hydration of fly ash and optimize the internal structure of the binder sand.

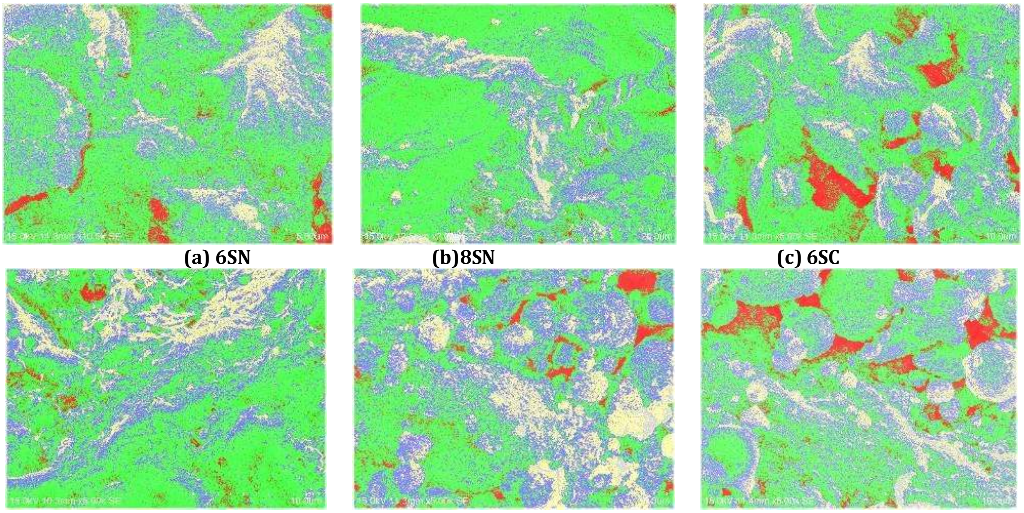

(a) 6SN (b)8SN (c) 6SC

(d) 8SC (e) 6FC (f) 8FC

Fig 15. Pore phase distribution of slag system and fly ash system

Table 4 Characteristic parameters of hole structure

sample	Porosity /%	Number of pores per unit/ piece	Maximum pore diameter/μ m	Total pore area /mm2
6SN	35.83	4456	2.325	0.0105
8SN	25.12	3598	0.206	0.0572
6SC	46.06	4140	3.028	0.0194
8SC	31.50	2712	0.726	0.0158
6FC	45.59	3618	2.854	0.0217
8FC	48.68	5685	3.624	0.0192

Conclusions

(1) Under the action of alkaline exciters, the initial and final setting time of slag system was substantially reduced compared with that of fly ash system, and the ce-mented sand strength of slag system was higher than that of fly ash system, in which slag showed a stronger sensitivity to NaOH and fly ash showed a stronger sensitivity to $Ca(OH)_2$.

(2) From the SEM, XRD, FTIR and AMR analyses, it can be seen that the choice of suitability of alkaline exciters is the key to determine the performance of the solid waste system, and the slag system has a more adequate hydration reaction under the action of 8% NaOH, generating more hydration products, optimizing the pore struc-ture, and significantly improving the mechanical properties. In contrast, the fly ash system showed higher sensitivity to $Ca(OH)_2$. At 6% $Ca(OH)_2$ doping, the hydration reaction of fly ash was more adequate, the polymerization of hydration products was higher, the pore structure was optimized significantly, and the mechanical properties were optimal. Excessive $Ca(OH)_2$ would inhibit the hydration reaction of fly ash and lead to strength deterioration. In addition, it can be clearly observed from the SEM images that fly ash is easy to generate reticulated C-(A)-S-H gel, N-A-S-H gel with 8% $Ca(OH)_2$ and flocculated C-(A)-S-H gel, N-A-S-H gel with 6% $Ca(OH)_2$.

(3) The pore characteristics and distribution of the slag system under NaOH exci-tation and the fly ash system under $Ca(OH)_2$ excitation were more reasonable, in which the pore structure of

Title

The title should be rephrased, fly ash and slag should appear in the title

Abstract

The abstract lacks quantity includes results obtained for compressive strength and flexural strength. Ensure that you contrast between results obtained for slag and fly ash.

Rewrite the abstract to improve readability

Correct misspelt words

Introduction

The justification for the study is not well established. Improve on this

Rephrase sentence: is China's major contributor to emissions

Use fullstop (.) before The

Start as a new paragraph

Methods

Improve this paragraph to aid coherency. Also provide pictures of the materials used. It is important to provide the particle size distribution of the slag and the fly ash.

Provide detailed information about this procedures. Just as was done for compressive strength. Describe all the testing methods to a great detail in different subsections. XRD, FTIR, TAM, BSE

Results

What do you mean by change rule diagram

The sentence is too long. Consider breaking the sentences to improve readability.

Improve readability. Discuss relative to existing literature

The method for sensitivity analysis was not captured in the methods

Highlight the important minerals and show the observable changes using inscribed rectangles.

FTIR analysis not FIRT Analysis

Introduce a shape here to call attention of the reader to the difference in intensity.

Improve the discussion by making reference to recent literature. (**Hydration characterization**)

Describe the method in detail under the method section. (**Pore physical phase distribution and pore structure characteristics**)

Consider rephrasing the sentence. Also improve this section with information from literature. What is the ideal pore area for geopolymer composites based on previous attempts by other researchers.

Conclusion

Provide a brief information about the purpose of the study before itemising the conclusions. Also, include the limitations of this study after the conclusion. Furthermore, I will suggest you give the entire manuscript to a native English speaker to improve the grammar, punctuation and sentences. There are a lot of grammatical inconsistency
