# Supplementary material for: Hydration product phase evolution and mortar strength development in alkali-activated slag and fly ash systems
Source: PLoS One. 2025 Dec 4;20(12):e0338119. doi: 10.1371/journal.pone.0338119 (PMC12677521; doi:10.1371/journal.pone.0338119)
Supplement: Attachment — Submitted filename: Reviewer 2.doc [file pone.0338119.s002.doc]

Dear editors and reviewers：

We are deeply grateful for your letter and the valuable feedback from the reviewers on our manuscript titled "Study on the Phase Evolution of Hydration Products in Alkali-Activated Solid Waste Materials and Mortar Strength" (PONE-D-25-18472). These comments have been very constructive in enhancing the quality of the manuscript. Based on the reviewers' suggestions, we have made comprehensive revisions to the manuscript, with all changes highlighted in red. We hope that the revised manuscript meets the publication standards of your journal.

The reviewers' comments are highlighted in italics, with specific issues numbered. We have responded to each of these issues individually, presented in regular font and highlighted in red. We have made every effort to improve the manuscript and sincerely thank the editors and reviewers for their hard work. We hope that the revised manuscript will be recognized.

Thank you again for your comments and suggestions.

Yours Sincerely,

Pro. Aimin Gong

College of Hydraulic EngineeringYunnan Agricultural University

E-mail: [13708457658@163.com](mailto:13708457658@163.com)

**Title**

**Comments 1:** *The title should be rephrased, fly ash and slag should appear in the title.*

**Response 1:** We sincerely appreciate your suggestion! The manuscript title has been revised to: “Study on the Hydration Product Phase Evolution and Mortar Strength of Alkali-Activated Slag and Fly Ash Systems”.

**Abstract**

**Comments 1:***The abstract lacks quantity, include results obtained for compressive strength and flexural strength. Ensure that you contrast between results obtained for slag and fly ash.*

*Rewrite the abstract to improve readability。*

*Correct misspelt words*

**Response 1:** Thank you for your comments. We have revised the abstract to add the results of compressive strength and flexural strength tests, clarify the difference in strength between slag and fly ash under alkali excitation, and modify the content as indicated in red on lines 26-29 of page 2. Spelling errors have also been corrected

**Introduction**

**Comments 1:** *The justification for the study is not well established. Improve on this Rephrase sentence: is China's major contributor to emissions.*

**Response 1:** Thank you for your suggestions. In the Introduction section, we have supplemented the background and significance of this study, adding a detailed discussion on alkali-activated geopolymer cementitious materials as a green alternative to cement. The revised content is marked in red annotations in lines 37–47 on pages 2–3 (Introduction section).Of course, as a substitute for cement, its engineering applications require further in-depth exploration and discussion. Additionally, the wording throughout the text has been revised.

**Comments 2:** *se fullstop (.) before The Start as a new paragraph。*

**Response 2:** We fully accept your suggestions and have revised the relevant sections accordingly.

Methods

**Comments 1:** *Improve this paragraph to aid coherency. Also provide pictures of the materials used. It is important to provide the particle size distribution of the slag and the fly ash.*

**Response 1:** We fully endorse this comment and have supplemented the manuscript with micrographs of the raw materials employed, as shown in Fig. 1. Additionally, the particle size distribution ranges of slag and fly ash have been incorporated into the materials description section. These revisions are highlighted in red on page 5, lines 99-102.

**Comments 2:** *Provide detailed information about this procedures. Just as was done for compressive strength. Describe all the testing methods to a great detail in different subsections. XRD, FTIR, TAM, BSE*

**Response 2:** We sincerely appreciate your thorough suggestions! The testing methods, including XRD, FTIR, TAM, and BSE, have been described in detail in the specimen preparation and testing sections. The change can be seen in red on pages 7 to 9, lines 126-174.

Results

**Comments 1:** *What do you mean by change rule diagram.*

*The sentence is too long. Consider breaking the sentences to improve readability.*

**Response 1:** We sincerely apologize for the inaccurate terminology " rule diagram" and have revised it. The change can be seen in red on pages 9 to 10, line 178-179. Additionally, overly lengthy sentences throughout the manuscript have been streamlined for enhanced clarity.

**Comments 2:** *improve readability. Discuss relative to existing literature*

**Response 2:** Thank you for your valuable feedback. We have carefully reviewed the manuscript and made revisions to improve readability and strengthen the discussion with existing literature.

**Comments 3:** *The method for sensitivity analysis was not captured in the methods.*

**Response 3:** Thank you very much for your thorough review of the manuscript and your valuable feedback. During the revision process, we decided to remove the section on sensitivity analysis from the original manuscript. This decision was made to preserve the core focus and overall integrity of the article.

**Comments 4:** *Highlight the important minerals and show the observable changes using inscribed rectangles.*

**Response 4:** We recognize the merit of highlighting mineral phases as proposed. However, the current analytical methodology yields data at a scale where precise mineral boundary delineation remains technically challenging.

**Comments 5:** *FTIR analysis not FIRT Analysis*

*Introduce a shape here to call the reader to the difference in intensity.*

**Response 5:** We sincerely appreciate you careful reading and valuable feedback. The term "FIRT" in the section title has been corrected to "FTIR". FTIR analysis has supplemented the difference in strength of slag and fly ash hydration systems under alkaline excitation. See pages 20 to 21, lines 378 to 395 marked in red.

**Comments 6:** *Improve the discussion by making reference to recent literature. (Hydration characterization)*

**Response 6:** We sincerely appreciate your suggestion to improve the discussion by incorporating recent literature on hydration characterization. In response, we have expanded the discussion in Section The revised content can be found in the red-highlighted text on page 24, lines 435–541.

**Comments 7:** *Describe the method in detail under the method section. (Pore physical phase distribution and pore structure characteristics)*

**Response 7:** Thank you for this valuable suggestion. As requested, we have added a detailed paragraph in the Methods section elaborating on the experimental procedures. highlighted in red on page 9, lines 164-167.

**Comments 8:** *Consider rephrasing the sentence. Also improve this section with information from literature. What is the ideal pore area for geopolymer composites based on previous attempts by other researchers?*

**Response 8:** We sincerely appreciate your suggestions. We have revised the sentences with problems in the paper's expression. Furthermore we analyzed the changes in pore number, maximum pore size, and total pore area. See pages 22 to 23, lines 400-417 marked in red, and point (3) in the "Conclusion".

Conclusion

**Comments 1:** *Provide a brief information about the purpose of the study before itemising the conclusions. Also, include the limitations of this study after the conclusion. Furthermore, I will suggest you give the entire manuscript to a native English speaker to improve the grammar, punctuation and sentences. There are a lot of grammatical inconsistency.*

**Response 1:** We fully endorse this recommendation. The opening paragraph of the Conclusion section now explicitly states the research objectives, while the concluding segment has been supplemented with the study's limitations. Additionally, comprehensive refinements have been made to grammar, punctuation, and sentence structure throughout.
