# Supplementary material for: Hydration product phase evolution and mortar strength development in alkali-activated slag and fly ash systems
Source: PLoS One. 2025 Dec 4;20(12):e0338119. doi: 10.1371/journal.pone.0338119 (PMC12677521; doi:10.1371/journal.pone.0338119)
Supplement: Attachment — Submitted filename: Reviewer #4.docx [file pone.0338119.s003.docx]

Dear editors and reviewers：

We are deeply grateful for your letter and the valuable feedback from the reviewers on our manuscript titled " Study on the Hydration Product Phase Evolution and Mortar Strength of Alkali-Activated Slag and fly ash systems" (PONE-D-25-18472R1). These comments have been very constructive in enhancing the quality of the manuscript. Based on the reviewers' suggestions, we have made comprehensive revisions to the manuscript, with all changes highlighted in red. We hope that the revised manuscript meets the publication standards of your journal.

The reviewers' comments are highlighted in italics, with specific issues numbered. We have responded to each of these issues individually, presented in regular font and highlighted in red. We have made every effort to improve the manuscript and sincerely thank the editors and reviewers for their hard work. We hope that the revised manuscript will be recognized.

Thank you again for your comments and suggestions.

Yours Sincerely,

Pro. Aimin Gong

College of Hydraulic Engineering Yunnan Agricultural University

E-mail: [13708457658@163.com](mailto:13708457658@163.com)

**Reviewer** **#4:**

**Comments 1:** *The manuscript is poorly written and presented. I am unable to read the manuscript. All aspects of writing in English are completely ignored. Perhaps the authors can get assistance in English writing. One can not follow exactly how the experimental work was carried out.*

**Response 1:** We wish to express our gratitude for the reviewer's comments regarding the language and presentation of our manuscript. These suggestions have been duly incorporated to improve the clarity and readability of the paper.
