# Supplementary material for: Hydration product phase evolution and mortar strength development in alkali-activated slag and fly ash systems
Source: PLoS One. 2025 Dec 4;20(12):e0338119. doi: 10.1371/journal.pone.0338119 (PMC12677521; doi:10.1371/journal.pone.0338119)
Supplement: Attachment — Submitted filename: Reviewer_#4_auresp_3.docx [file pone.0338119.s004.docx]

Dear Editors and Reviewers,

We sincerely thank you for your letter and the valuable comments from the reviewers on our manuscript titled " Hydration Product Phase Evolution and Mortar Strength Development in Alkali-Activated Slag and Fly Ash Systems" (PONE-D-25-18472R2). These suggestions have been highly instructive in improving the quality of our paper. Based on the reviewers' feedback, I have thoroughly revised the manuscript, with all changes highlighted in red. We hope that the revised version meets the publication standards of your journal.

The reviewers' comments are presented in italics, with specific issues numbered. I have addressed each of these points individually, and my responses are provided in standard font and highlighted in red. I have made every effort to improve the manuscript and deeply appreciate the hard work of the editors and reviewers. The successful publication of this research is crucial for the completion of my degree application, and I sincerely hope that the revised manuscript will be accepted.

Thank you once again for your comments and suggestions.

Sincerely,

(Student's Name)

Supervised by: Professor Aimin Gong

College of Hydraulic Engineering, Yunnan Agricultural University

Email: 13708457658@163.com

**Reviewer** **#4:**

**Comments 1:** *The manuscript has adequate data that is presentable for publication. However, the manuscript still has been written in poor English. Methodology has improved but will still require to be worked on.Results and discussion is poorly done. There is no scientific presentation of the results and discussion.*

**Response 1:** Thank you for your positive assessment of the manuscript's data. We sincerely apologize for the deficiencies in English language and scientific presentation in the previous version. In response, we have conducted a comprehensive revision of the manuscript. Furthermore, we have reorganized the "Results and Discussion" section to enhance the logical flow of the scientific narrative and ensure a clearer and more persuasive presentation of the findings. All modifications have been highlighted in red for your convenience.
