# Supplementary material for: Hydration product phase evolution and mortar strength development in alkali-activated slag and fly ash systems
Source: PLoS One. 2025 Dec 4;20(12):e0338119. doi: 10.1371/journal.pone.0338119 (PMC12677521; doi:10.1371/journal.pone.0338119)
Supplement: Attachment — Submitted filename: Reviewer 1.doc [file pone.0338119.s005.doc]

Dear Reviewer,

We sincerely appreciate your valuable comments on our manuscript titled "Study on the Phase Evolution of Hydration Products and the Strength of Mortar in Alkali-Activated Solid Waste Materials" (PONE-D-25-18472R3). We are especially grateful for the insightful suggestions provided throughout multiple rounds of revision, which have deepened my understanding of academic rigor and significantly contributed to improving the quality of this paper. In response to your feedback, we have thoroughly revised the manuscript, with all changes highlighted in red.

The reviewer’s comments are presented in italics, and our responses are provided in regular font, also highlighted in red. We have made every effort to refine the content of the paper and sincerely thank you for your diligent work. We hope that the revised manuscript meets with your approval.

Thank you again for your comments and suggestions.

Yours Sincerely,

Pro. Aimin Gong

College of Hydraulic Engineering Yunnan Agricultural University

E-mail: [13708457658@163.com](mailto:13708457658@163.com)

**Reviewer1：**

**Comments :** *After the following minor revision, the paper can be moved toward next step.*

*1. Reference format issues. For example, 11, 24, 25, , … [J/OL]!!!, …*

*2. Remove the Chiness characters*

*3. Writing Styles, some with all capital letters, some not, …*

*4. Some references like 7, 8, 14, 28, … don’t have any authors!!!!*

*5. Some references don’t have any DOI, like 5, …*

*6. Use DOI link (https://doi.org/...)*

**Response :** Thank you for your valuable comments and suggestions on our manuscript. We have carefully revised the paper according to your feedback and have highlighted all changes in red within the text. The specific modifications are as follows:

1.Reference Format: We have thoroughly checked and standardized the reference format. Irregular identifiers such as [J/OL] in entries (e.g., 11, 24, 25, etc.) have been corrected.

2.Removal of Chinese Characters: All non-essential Chinese characters have been completely removed from the manuscript.

3.Writing Styles: Inconsistencies in capitalization have been corrected to ensure consistency throughout the manuscript.

4.Missing Authors: Author information missing from reference entries (e.g., 7, 8, 14, 28, etc.) has been added.

5.Addition of DOIs: Digital Object Identifiers (DOIs) have been added for all applicable references (including entry 5, etc.).

6.DOI Links: The links for all references with DOIs have been uniformly formatted to the https://doi.org/... style.
